# The two most common histological subtypes of malignant germ cell tumour are distinguished by global microRNA profiles, associated with differential transcription factor expression

**DOI:** 10.1186/1476-4598-9-290

**Published:** 2010-11-08

**Authors:** Matthew J Murray, Harpreet K Saini, Stijn van Dongen, Roger D Palmer, Balaji Muralidhar, Mark R Pett, Matias Piipari, Claire M Thornton, James C Nicholson, Anton J Enright, Nicholas Coleman

**Affiliations:** 1 Medical Research Council Cancer Cell Unit, Cambridge, CB2 0XZ, UK; 2 EMBL-European Bioinformatics Institute, Hinxton, Cambridge, CB10 1SD, UK; 3 Wellcome Trust Sanger Institute, Hinxton, Cambridge, CB10 1SA, UK; 4 Department of Pathology, Royal Group of Hospitals Trust, Belfast, UK; 5 Department of Paediatric Haematology and Oncology, Addenbrooke's Hospital, Cambridge, CB2 0QQ, UK; 6 Department of Pathology, University of Cambridge, CB2 1QP, UK

## Abstract

**Background:**

We hypothesised that differences in microRNA expression profiles contribute to the contrasting natural history and clinical outcome of the two most common types of malignant germ cell tumour (GCT), yolk sac tumours (YSTs) and germinomas.

**Results:**

By direct comparison, using microarray data for paediatric GCT samples and published qRT-PCR data for adult samples, we identified microRNAs significantly up-regulated in YSTs (n = 29 paediatric, 26 adult, 11 overlapping) or germinomas (n = 37 paediatric). By Taqman qRT-PCR we confirmed differential expression of 15 of 16 selected microRNAs and further validated six of these (miR-302b, miR-375, miR-200b, miR-200c, miR-122, miR-205) in an independent sample set. Interestingly, the miR-302 cluster, which is over-expressed in all malignant GCTs, showed further over-expression in YSTs versus germinomas, representing six of the top eight microRNAs over-expressed in paediatric YSTs and seven of the top 11 in adult YSTs. To explain this observation, we used mRNA expression profiles of paediatric and adult malignant GCTs to identify 10 transcription factors (TFs) consistently over-expressed in YSTs versus germinomas, followed by linear regression to confirm associations between TF and miR-302 cluster expression levels. Using the sequence motif analysis environment iMotifs, we identified predicted binding sites for four of the 10 TFs (GATA6, GATA3, TCF7L2 and MAF) in the miR-302 cluster promoter region. Finally, we showed that miR-302 family over-expression in YST is likely to be functionally significant, as mRNAs down-regulated in YSTs were enriched for 3' untranslated region sequences complementary to the common seed of miR-302a~miR-302d. Such mRNAs included mediators of key cancer-associated processes, including tumour suppressor genes, apoptosis regulators and TFs.

**Conclusions:**

Differential microRNA expression is likely to contribute to the relatively aggressive behaviour of YSTs and may enable future improvements in clinical diagnosis and/or treatment.

## Background

Germ cell tumours (GCTs) are clinico-pathologically complex neoplasms that arise from early infancy through to late adulthood [1]. Malignant GCTs are classified as germinomas (collectively referring to testicular seminoma, ovarian dysgerminoma and extragonadal germinoma) and non-germinomatous tumours, which include yolk sac tumours (YSTs) [1]. Germinomas and YSTs are the most common pure histological subtypes of malignant GCT.

Although treatment of most malignant GCTs is successful, there are still patient groups with a less favourable outcome. For example, considering intracranial malignant GCTs treated with radiotherapy alone, five-year overall survival is well in excess of 90% for germinomas [2], but < 50% for non-germinomatous tumours, with many early relapses [3]. Even adding systemic chemotherapy for the latter group produces a five-year relapse-free survival of only 67% [4], remaining substantially worse than the intracranial germinoma group. Likewise, outcomes in extracranial non-germinomatous tumours are inferior to germinomas, in both paediatric and adult patients [5,6].

An important step towards improving outcomes for patients with unfavourable malignant GCTs is to identify biological differences between the principal histological subtypes, as these may account for the observed differences in clinical behaviour and treatment response. In previous work, we systematically determined expression of mRNA and microRNAs in a large group of paediatric malignant GCT samples [7,8] and compared our data with available findings for adult cases [9,10]. When comparing YSTs versus germinomas, mRNA profiles differed primarily by histological subtype but also by patient age (paediatric versus adult) [7]. Germinomas recapitulated an undifferentiated and pluripotent phenotype, over-expressing the embryonic stem cell (ESC) markers *NANOG, POU5F1 *(*OCT3/4*) and *UTF1*, whereas YSTs displayed extra-embryonic differentiation while maintaining a proliferative phenotype [7].

Recently, we performed microarray-based global microRNA analysis in paediatric malignant GCTs, combined with re-assessment of reverse-transcription PCR (qRT-PCR) microRNA profiling of adult cases [9]. MicroRNAs (miR-) are short, non-protein coding RNAs that regulate gene expression via translational repression and/or mRNA degradation. We demonstrated that all malignant GCTs co-ordinately over-express the miR-371~373 and miR-302 microRNA clusters [compared to a combined non-malignant control group of normal gonadal samples and benign GCTs (teratomas)], regardless of patient age, tumour histological subtype or anatomical site [8].

In the present study, we have analysed our data further, to test the hypothesis that the two most common pure malignant GCT subtypes, YSTs and germinomas, differentially express sets of microRNAs that contribute to the observed clinico-pathological differences. Our study had four principal aims. First, we sought to identify differences in the microRNA microarray profiles of paediatric YSTs versus germinomas, examining tissues of both gonadal and extragonadal origin. Second, we compared these findings with available qRT-PCR microRNA profiling data for adult gonadal YSTs and germinomas [9]. Third, we sought to confirm significant microRNA microarray findings by Taqman qRT-PCR in the same cohort of clinical samples, before selecting a panel of microRNAs to validate in an independent sample set. Finally, we used an integrative approach to identify transcription factors (TFs) that may be responsible for the observed differential microRNA profiles. We combined linear regression analysis of microRNA and mRNA expression levels in matched clinical samples with the motif scanning algorithm [11] integrated in the graphical motif analysis environment iMotifs [12]. This algorithm predicts TF binding site motif matches in gene promoter regions, resulting in individual motif bit scores for each match and an overall empirical *e*-value for each TF.

Together, these analyses have identified robust microRNA expression differences between YSTs and germinomas, which may substantially contribute to observed differences in disease natural history and offer the potential for improving differential diagnosis and treatment selection.

## Methods

### Tumour samples

**Figure 1:**
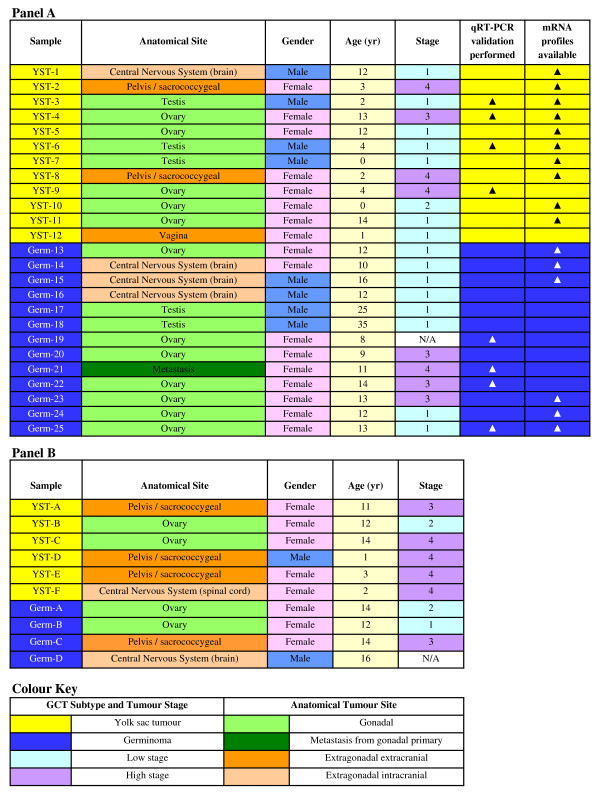
**Clinico-pathological data for the paediatric malignant GCTs analysed**. Panel A shows the 25 cases assessed by microarray, while Panel B shows the 10 independent cases analysed by qRT-PCR.

The study received Multicentre Research Ethics Committee (ref: 02/4/071) and Local Research Ethics Committee (ref: 01/128) approval. For microarray analysis and initial data validation we studied 25 samples, each from a different patient. These represented 23 paediatric GCTs (12 YSTs, 11 germinomas), plus two testicular germinomas from young adults [8], as such tumours are extremely rare in the paediatric age-range. To avoid confusion with data from our re-assessment of microRNA expression in adult YSTs and germinomas (see below), both of the young adult samples are hereafter referred to as 'paediatric'. For both YSTs and germinomas, samples were included from male and female patients and from gonadal and extragonadal (including intracranial) sites. Further clinico-pathological details are provided in Figure 1, Panel A. All samples, including those derived from mixed GCTs, were completely or predominantly (>90%) composed of a single malignant element. Further validation of differential microRNA expression was performed in an independent set of 10 paediatric tumour samples (six YSTs, four germinomas), selected to represent a mixture of male and female patients and gonadal and extragonadal (including intracranial) sites (Figure 1, Panel B).

### microRNA microarray expression profiling

Total RNA was isolated as described previously [7]. Sample and human reference RNA were hybridized to the miRCURY LNA array platform (Exiqon, Vedbaek, Denmark), as described [8]. Data files were updated to miRBase v13.0, which annotated 615 microRNA probes, and analysed using Bioconductor in the statistical software environment *R *[8]. Raw data for these samples is available at the Gene Expression Omnibus [GEO accession number GSE18155]. MicroRNAs with adjusted *p*-values < 0.01 [13] were considered to be differentially expressed, while heatmaps were generated from the most significantly differentially expressed microRNAs (adjusted *p *< 1 × 10^-5^).

We compared our findings for the paediatric samples with published qRT-PCR expression data for microRNAs in adult gonadal YSTs (n = 8) and germinomas (n = 25) [9]. Raw cycle threshold (CT) data were downloaded and data analysis performed using Bioconductor in *R*, as described [8]. We obtained *ΔΔC*T values, which were used to perform supervised hierarchical clustering analysis and identify differentially expressed genes, defined as for the paediatric samples.

### Taqman qRT-PCR validation of microRNA microarray levels

MicroRNA expression changes detected by microarray in YSTs versus germinomas were first confirmed in a randomly selected subset of eight tumour samples (four YSTs, four germinomas; see Figure 1, Panel A), using Taqman microRNA assays (Applied Biosystems), according to the manufacturer's instructions. Relative amounts of 16 selected microRNAs were determined using the *ΔΔC*T method, normalized to RNU24, which showed the least variation between the eight samples analyzed of four small nuclear and nucleolar housekeeping genes tested (RNU6b, RNU24, RNU38b and RNU43; data not shown). For each microRNA, expression values were referenced to the sample with the lowest normalized expression levels, as previously described [8,14].

Six of the 16 microRNAs were selected for further validation in an independent set of 10 tumour samples (Figure 1, Panel B), and for quantification in total RNA from normal human ovary and testis (both Ambion). The six microRNAs were normalized to RNU24 and expression levels referenced to Universal Human Reference total RNA (Stratagene). Expression differences between the YST and germinoma group were assessed using a two-sided Welch's (unequal variance) *t*-test [15], with *p*-values ≤ 0.05 considered to be significant.

### mRNA expression analysis

Matching global mRNA expression profiles were available for 16 of the 25 paediatric malignant GCTs examined by microRNA microarray (10 YSTs, six germinomas; see Figure 1, Panel A). Profiling had previously been performed using the HG-U133A GeneChip (Affymetrix, Santa Clara, CA), which contained 22,283 probe sets corresponding to 13,042 genes [GEO accession number 10615; [7]]. In addition, we re-analysed published data from a study of adult testicular malignant GCTs (TGCTs) that used the same microarray platform [GEO accession number GSE3218; [10]], excluding two suboptimal YST samples (K14 and K18) [7]. We re-analysed data from 20 suitable TGCTs, representing eight pure YSTs and 12 pure germinomas. Raw mRNA (.CEL) files were processed, normalized and analyzed, using the Affymetrix annotation of March 2009, as described [8]. Genes with log_2 _fold-change >1.5 and adjusted *p *< 0.01 were classified as differentially expressed.

### Transcriptional regulation of differentially expressed microRNA clusters

We interrogated available mRNA expression profiles for paediatric and adult malignant GCTs [8,10] to identify up-regulated TFs that may be responsible for the increased expression of the miR-302 cluster in YSTs versus germinomas. For this screening exercise, we applied less stringent criteria of log_2 _fold-change >1.0 and adjusted *p *< 0.01. For all TFs so identified, we performed linear regression analysis of the 16 paediatric samples (10 YSTs, six germinomas) for which matched microRNA and mRNA expression data were available (Figure 1, Panel A), plotting TF levels against median expression of the main members of the miR-302 cluster. *P*-values ≤ 0.05 were considered significant.

### Motif scanning

We next explored whether TFs that were differentially expressed between YSTs and germinomas had computationally predicted binding sites in the promoter and upstream regions of the miR-302 cluster. To do this we utilized the sequence motif analysis environment iMotifs [12], which allows visualisation of nucleotide sequences and identifies motif matches within promoter sequences, using the scanning algorithm included in the NestedMICA suite [11].

We analysed the 10 kb region upstream of, and including, the miR-302 cluster. This was chosen as a conservative distance over which a TF may exert its influence, as previously microRNAs within 50 kb of each other have been shown to be transcriptionally co-regulated [16,17]. The 10 kb sequence was downloaded from the Ensembl database (coordinates 113,569,030 to 113,579,030; corresponding to the NCBI37 human gene assembly) [18], repeat-masked http://www.repeatmasker.org/ and dusted [19] to remove low-complexity repeats. TF binding motifs were obtained from the TRANSFAC database [20], version 12.2, and predicted binding sites within the 10 kb sequence were identified using the NestedMICA suite [11], which yields normalised motif bit scores for each predicted site. As the motif bit score distribution of individual motifs varied depending on both nucleotide length (number of columns) and information content, a bit score significance threshold was determined individually for each motif. This was calculated by subdividing the motif bit scores to 1-bit intervals, and testing for over-representation of high-scoring motif hits at high-scoring intervals when compared to the sequence background model (*p *< 0.05; binomial test), as described [11].

As an additional measure, a single empirical *e*-value was derived for each TF computationally predicted to bind to the miR-302 promoter region. These values were computed from the maximum bit scores (i.e. values closest to zero) achieved by each of the TF motifs of interest that had at least one significant match in the 10 kb sequence region. The *e*-value estimation was performed by shuffling the input sequence 100,000 times and counting the frequency at which the shuffled sequence achieved a score that was equal to, or better than, the observed bit score. *E*-values < 0.05 were considered to be significant.

## Results

### Identification of microRNAs differentially expressed in YST versus germinomas

**Figure 2:**
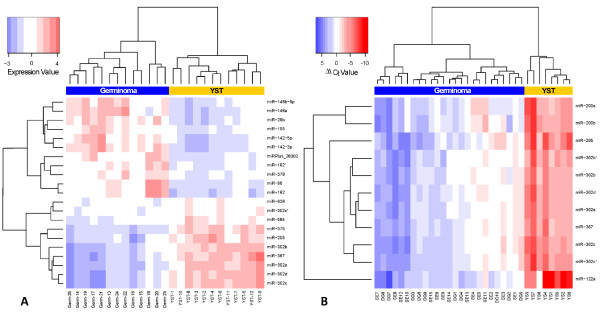
**Supervised hierarchical clustering analysis of YSTs versus germinomas for A) paediatric and B) adult tumours**. Analysis was based on the most significantly differentially expressed microRNAs (adjusted* p *< 1 × 10^-5^).

From our microarray analysis we identified 66 microRNAs that were significantly differentially expressed in paediatric YSTs versus germinomas. Of these, 29 were over-expressed in YSTs (43.9%) and 37 over-expressed in germinomas (56.1%) (Table 1). The fold changes for the former were generally greater than for the latter, with 12/29 (41.4%) microRNAs over-expressed in YSTs having log_2 _fold changes >2, compared with only 8/37 (21.6%) in germinomas. The most significantly differentially expressed microRNAs (*p *< 1 × 10^-5^; n = 21) robustly discriminated between the two tumour types on hierarchical clustering analysis (Figure 2A). Interestingly, these microRNAs included all main members of the miR-302 cluster (miR-302a~302d and miR-367), which although over-expressed in all malignant GCTs compared to non-malignant tissues [8], were further over-expressed in YSTs compared to germinomas. Indeed, six of the top eight ranked microRNAs over-expressed in YSTs belonged to this single cluster. Other significantly over-expressed microRNAs in paediatric YSTs, with large log_2 _fold changes, included miR-375 (log_2 _fold change 3.22), miR-205 (3.25), miR-122 (3.71), miR-200a~200c cluster (2.28, 2.56 and 2.42) and miR-141 (2.06) (Table 1). MicroRNAs significantly over-expressed in paediatric germinomas included miR-146a (log_2 _fold change 2.94), miR-142-3p/5p (2.97 and 2.73), miR-182 (2.64), miR-96 (2.43) and miR-29a~b (2.40 and 2.67) (Table 1).

**Table 1:** Significantly differentially expressed microRNAs in YSTs versus germinomas for paediatric tumours

Over-expressed in YSTs	Log_2 _fold change	Adjusted *p*-value	Over-expressed in germinomas	Log_2 _fold change	Adjusted *p*-value
miR-375	3.22	2.61E-15	miR-182*	1.90	1.81E-10

**miR-302a**	**4.17**	**6.61E-13**	miR-146a	2.94	6.54E-08

**miR-302c**	**4.24**	**1.53E-10**	miR-142-5p	2.73	4.23E-07

**miR-367**	**4.12**	**3.73E-10**	miR-142-3p	2.97	4.23E-07

**miR-302d**	**4.11**	**1.07E-09**	miR-182	2.64	4.90E-07

miR-302c*	**1.35**	**1.85E-07**	miR-96	2.43	5.09E-07

miR-584	1.64	4.23E-07	miRPlus_28302	1.91	9.46E-07

**miR-302b**	**3.49**	**1.02E-06**	miR-146b-5p	2.26	9.46E-07

miR-205	3.25	1.78E-06	miR-155	1.84	1.02E-06

miR-638	1.21	9.52E-06	miR-29b	2.67	4.55E-06

miR-2110	0.94	1.71E-05	miR-378	1.54	8.72E-06

miR-30b*	1.15	5.28E-05	miR-30e	1.72	4.30E-05

miR-122	3.71	1.21E-04	miR-183	1.54	1.21E-04

miR-518e*	0.85	1.34E-04	miR-520b	1.65	1.21E-04

miR-572	1.21	1.84E-04	miR-29a	2.40	3.02E-04

miR-200b	2.56	3.17E-04	miR-101	1.50	3.38E-04

miRPlus_27560	1.18	3.17E-04	miR-520c-3p	1.35	3.53E-04

miR-766	0.85	3.17E-04	miR-25	1.06	4.58E-04

miR-940	0.98	5.78E-04	miR-342-3p	1.47	4.58E-04

miR-200c	2.42	6.66E-04	miR-590-5p	0.97	6.44E-04

miR-483-3p	0.84	1.47E-03	miR-526b*	1.23	7.91E-04

miR-200a	2.28	1.85E-03	miR-520g	1.44	8.73E-04

miR-455-3p	1.33	2.01E-03	miR-135b	1.66	1.50E-03

miR-296-5p	0.69	2.17E-03	miR-515-5p	1.61	1.58E-03

miR-602	0.83	2.59E-03	miR-30a	1.22	2.37E-03

miR-720	0.83	3.76E-03	miR-9	1.77	2.37E-03

miR-409-3p	1.18	5.85E-03	miR-29c*	0.78	3.64E-03

miR-210	0.74	6.36E-03	miR-29c	1.56	3.64E-03

miR-141	2.06	8.90E-03	miR-520g/h	1.13	3.76E-03

			miR-512-3p	1.10	3.81E-03
			
			let-7i	1.38	3.95E-03
			
			miR-34a	1.03	4.08E-03
			
			miR-340	0.68	4.87E-03
			
			miR-373	1.56	5.53E-03
			
			miR-515-3p	0.98	7.06E-03
			
			miR-32*	0.75	8.18E-03
			
			miR-151-3p	0.80	8.29E-03

Analysis of the paediatric microarray data resulted in the log_2 _fold changes shown. microRNAs are ranked by adjusted *p*-value. Members of the miR-302 cluster are shown in bold.

Re-analysis of the published adult GCT qRT-PCR data [9] identified 26 microRNAs that were differentially expressed between YSTs and germinomas (Table 2), with all showing over-expression in YSTs. Although only 17 of the 37 microRNAs identified as over-expressed in paediatric germinomas by microarray analysis were present on the 156 microRNA Taqman platform employed for the adult study, it was surprising that no microRNA in this adult dataset showed over-expression in germinomas. Nevertheless, the most significantly differentially expressed microRNAs in the adult comparison (*p *< 1 × 10^-5^; n = 11) completely distinguished YSTs from germinomas on hierarchical clustering (Figure 2B). Moreover, the miR-302 cluster was again significantly over-expressed in adult YSTs, with seven family members in the top 11 ranked differentially expressed microRNAs, a point not previously highlighted [9]. Other microRNAs over-expressed in both paediatric and adult YSTs included miR-205, miR-122 (miR-122a in the adult study, re-annotated as miR-122 in miRBase v13.0) and the miR-200a~200c family (Tables 1 and 2). It should be noted that miR-375, the top ranking differentially expressed microRNA in the paediatric dataset (up-regulated in YSTs), was not present on the Taqman platform.

**Table 2:** Significantly differentially expressed microRNAs in YSTs versus germinomas for adult tumours

Over-expressed in YSTs	ΔΔ Ct	Adjusted *p*-value	Over-expressed in germinomas	ΔΔ Ct	Adjusted *p*-value
miR-122a	-9.98	5.66E-09			
			
miR-302c*	-6.98	3.40E-08			
			
miR-205	-7.34	1.20E-07			
			
miR-200a	-6.57	1.85E-07			
			
miR-200b	-6.33	3.76E-07			
			
**miR-302a**	-5.81	3.76E-07			
			
**miR-302d**	-5.75	3.76E-07			
			
miR-302b*	-5.75	4.80E-07			
			
**miR-302b**	-5.59	4.80E-07			
			
**miR-367**	-5.56	7.35E-07			
			
**miR-302c**	-6.43	7.99E-07			
			
miR-200c	-5.49	1.23E-05			
			
miR-203	-4.65	6.75E-05			
			
miR-34c	-3.66	3.62E-04			
			
miR-339	-3.00	3.62E-04			
			
miR-144	-3.38	4.91E-04			
			
miR-107	-2.71	4.91E-04			
			
miR-17-5p	-3.15	1.10E-03			
			
miR-34b	-3.37	1.25E-03			
			
miR-106a	-3.08	1.57E-03			
			
miR-133b	-2.86	6.06E-03			
			
miR-338	-2.53	6.06E-03			
			
miR-214	-2.10	6.06E-03			
			
miR-133a	-2.73	6.46E-03			
			
miR-129	-4.12	8.16E-03			
			
miR-23b	-1.85	8.16E-03			

Analysis of the adult qRT-PCR data resulted in the ΔΔCt values shown. microRNAs are ranked by adjusted *p*-value. Members of the miR-302 cluster are shown in bold.

### Validation of microRNA expression differences by Taqman qRT-PCR

**Figure 3:**
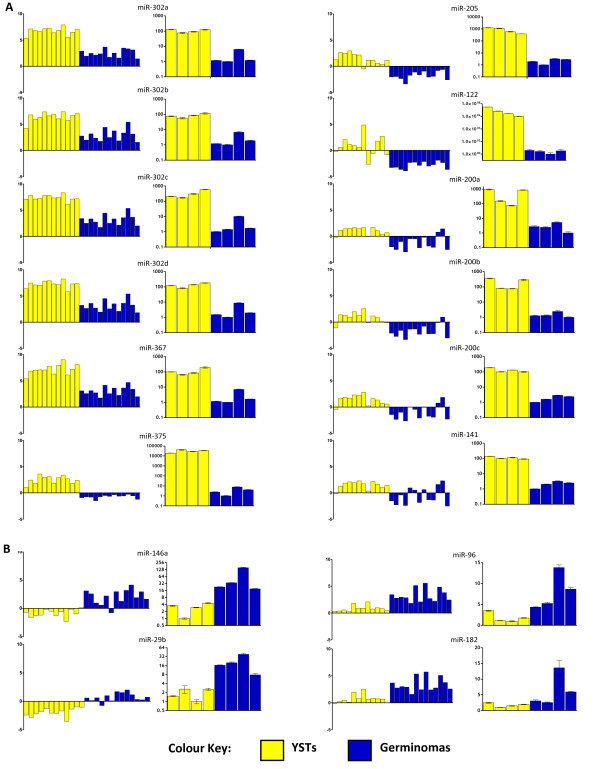
**Validation of microarray data by Taqman qRT-PCR**. Each pair of graphs relates to one of 16 selected microRNAs differentially expressed in YSTs versus germinomas, of which 12 were over-expressed in YSTs (A) and four over-expressed in germinomas (B). In each pair, the left hand graph shows microarray expression changes in the 25 paediatric tissue samples (12 YSTs, 13 germinomas), referenced to the median expression of eight control samples (fetal and normal gonad [8]). In all cases samples are in the order given in Figure 1, Panel A. Each right hand graph shows data from Taqman qRT-PCR expression analysis in eight tumours (four YSTs and four germinomas) representing a randomly selected subset of the 25 examined by microarray. qRT-PCR expression values are referenced to the sample with the lowest expression level. In all cases samples are in the order: YST-3, YST-4, YST-6, YST-9, Germ-19, Germ-21, Germ-22 and Germ-25.

We used qRT-PCR to confirm our microarray findings, examining a subset of eight of the 25 tumours analysed using microarrays (four YSTs, four germinomas; Figure 1, Panel A). We selected 16 for validation of the 66 microRNAs differentially expressed in paediatric YSTs versus germinomas. Of these, 12 were up-regulated in YSTs (across a range of observed fold changes and adjusted *p*-values) - all were confirmed as being over-expressed by qRT-PCR (Figure 3A). The remaining four microRNAs were up-regulated in germinomas, of which three were confirmed by qRT-PCR (Figure 3B).

**Figure 4:**
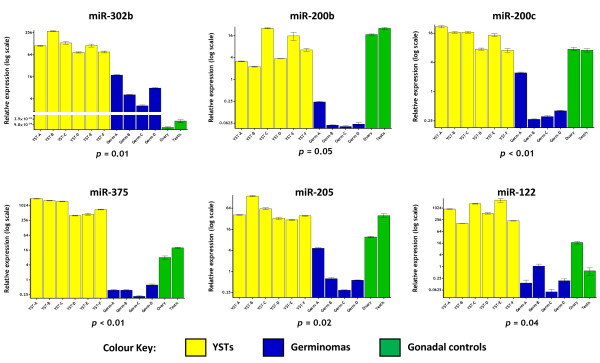
**Validation of microRNA expression changes in an independent sample set**. Six microRNAs over-expressed in YSTs were further quantified in an independent set of 10 paediatric tumours (six YSTs, four germinomas), plus normal gonadal (ovary and testis) samples, referenced to Universal Human Reference total RNA.

We next used an independent set of 10 malignant GCTs to confirm significant differential expression of six microRNAs selected from the group of 16. We chose microRNAs that were up-regulated in YSTs, as fold changes in YSTs were generally greater than in germinomas. We avoided multiple microRNAs from a single cluster, as the transcription of such microRNAs is co-ordinately regulated [16,17] and thus individual microRNAs are not independent of the others. Accordingly, the six microRNAs selected were each transcribed from an independent genomic locus, namely miR-375 (chromosomal location 2q35), miR-302b from the miR-302 cluster (4q25), miR-205 (1q32.2), miR-122 (18q21.31), miR-200b from the miR-200a~b cluster (1p36.33) and miR-200c from the miR-200c/miR-141 cluster (12p13.31) (Figure 4). We also quantified levels of these microRNAs in normal ovary and testis. We confirmed miR-302b over-expression in YSTs and (to a lesser extent) germinomas compared to gonadal control tissue, as previously identified by us [8]. Expression of miR-200b and miR-200c was similar in YSTs and gonadal tissue, but significantly down-regulated in germinomas. For miR-375, miR-205 and miR-122, expression in gonadal tissue lay between that for the over-expressing YSTs and under-expressing germinomas (Figure 4).

### Predicted transcriptional regulation of the miR-302 cluster

**Figure 5:**
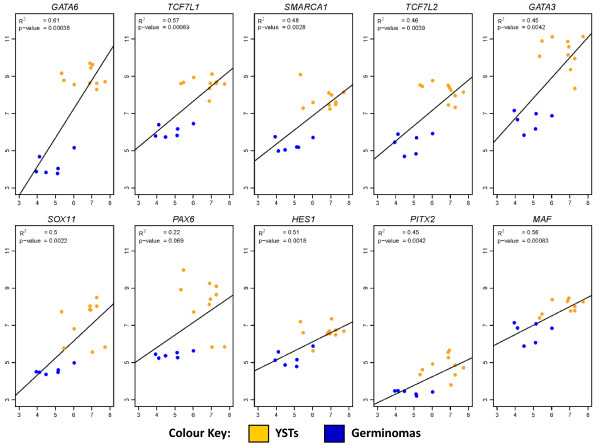
**Relationships between expression of the miR-302 cluster and transcription factors in YSTs and germinomas**. Each plot shows linear regression analysis for an individual transcription factor identified as over-expressed in both paediatric and adult YSTs (versus germinomas), using data from the 16 paediatric malignant GCT samples with matched mRNA and microRNA expression profiles. In each graph, mRNA levels for the transcription factor (*y*-axis) are plotted against the matched median expression value for the five main members of the miR-302 cluster, namely miR-302a~302d and miR-367 (*x*-axis).

We sought to identify candidate TFs responsible for the prominent over-expression of the miR-302 cluster in paediatric and adult YSTs versus germinomas. The miR-302 cluster is transcribed from the negative DNA strand at chromosome 4q25, with all the individual microRNAs sharing the same promoter region [21]. We first examined mRNA expression profiles in malignant GCTs, using our dataset for paediatric tumours [8] and published data for adult tumours [10]. We identified 10 candidate TFs that were significantly over-expressed in YSTs versus germinomas in both datasets, namely *GATA6, GATA3, TCF7L1, TCF7L2, SMARCA1, SOX11, PAX6, HES1, PITX2 *and *MAF *(Table 3). For these TFs, we next performed linear regression analysis using the 16 paediatric samples (10 YSTs, six germinomas) for which matched microRNA and mRNA expression data were available. We demonstrated a significant positive correlation (*p*≤ 0.05) between the median expression value for the five main microRNAs from the miR-302 cluster (miR-302a~d and miR-367) and expression levels of nine of the 10 TFs tested, with the correlation for the tenth TF, *PAX6*, approaching significance (*p *= 0.069). *GATA6 *was the top-ranked TF (p = 0.00038) in this comparison (Figure 5).

**Table 3:** Transcription factors up-regulated in YSTs versus germinomas

Transcription Factor		Paediatric Dataset		Adult Dataset		Motif Scanning		Gene Function
**Accession**	**Name**	**Rank** **(n = 594)**	**Log_2 _fold** **change**	**Rank** **(n = 730)**	**Log_2 _fold** **change**	**Maximum** **motif bit** **score**	***e*-value**	

NM_005257	***GATA6***	**20**	+4.72	**13**	+4.85	-0.28	**0.032**	Marker of early endodermal differentiation; transcriptional regulator of differentiation and proliferation

NM_031283	***TCF7L1***	**29**	+2.52	**36**	+2.17	-	**-**	Wnt pathway signalling

NM_003069	***SMARCA1***	**52**	+2.49	**52**	+3.27	-	**-**	Regulates transcription by altering chromatin structure. Involved in development and differentiation

NM_001146283	***TCF7L2***	**59**	+2.77	**64**	+3.09	-0.58	**0.184**	Wnt pathway signalling

NM_001002295	***GATA3***	**63**	+3.63	**146**	+3.66	-1.73	**0.173**	Marker of early endodermal differentiation; transcriptional regulator of differentiation and proliferation

NM_003108	***SOX11***	**229**	+2.62	**313**	+1.61	-	**-**	SRY (sex determining region Y)-box 11; embryonic development and cell fate; involved in tumorigenesis

NM_000280	***PAX6***	**316**	+2.77	**600**	+1.13	-	**-**	Paired box protein 6; marker of neuro-ectodermal differentiation

NM_005524	***HES1***	**355**	+1.41	**175**	+2.01	-	**-**	Hairy and enhancer of split 1; regulates growth and proliferation

NM_000325	***PITX2***	**356**	+1.41	**274**	+1.69	-	**-**	Paired like homeodomain 2; regulates terminal differentiation

NM_001031804	***MAF***	**365**	+1.39	**234**	+1.83	-3.93	**0.032**	Musculo-aponeurotic fibrosarcoma oncogene; involved in development and terminal differentiation

The 10 transcription factors significantly up-regulated in both paediatric and adult YSTs versus germinomas, including their log_2 _fold changes and ranks in the lists of differentially expressed mRNAs, are listed. The iMotifs values represent the maximum motif bit scores for each of the four individual transcription factors with predicted binding sites in the 10 kb miR-302 promoter region, together with derived empirical *e*-values. In iMotifs the optimal bit score is normalised to zero, with all other bit scores having negative values. Accordingly, the closer a score to zero, the greater is the significance of the predicted transcription factor binding site.

**Figure 6:**
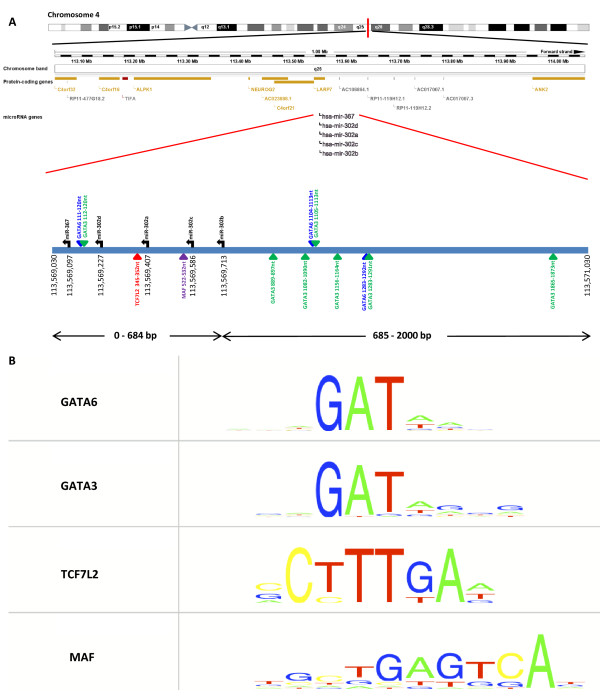
**Putative transcription factor binding sites in the miR-302 promoter region**. A) Schematic of the miR-302 genomic locus at chromosome band 4q25, adapted from [18], showing the 2 kb region upstream of the last microRNA in the cluster, miR-367. The miR-302 cluster itself is 684bp in length. Potential binding sites for four transcription factors (GATA6, GATA3, TCF7L2 and MAF) are shown by the coloured triangles. B) The consensus sequence binding motifs for the four transcription factors with predicted binding sites in the 10 kb region upstream of the miR-302 cluster. Nucleotide position is displayed horizontally and information content vertically. The relative height of the individual nucleotides represents the relative frequency of each nucleotide at that position, based on multiple sequence alignments obtained from known gene targets. Although the GATA6 binding motif is 10nt in length, the 10^th ^base has an extremely low information content and is not visible in the schematic.

Using iMotifs, we identified a total of 41 significant predicted binding sites for four of the 10 TFs in the 10 kb of sequence upstream of the distal microRNA in the miR-302 cluster, miR-367 (Additional File 1, Table S1 and Additional File 2, Figure S1). These TFs were GATA6 (9nt AAAGATAAG binding motif; 19 binding sites), GATA3 (9nt GAGATAGGG; 18 sites), TCF7L2 (8nt CCTTTGAA; 2 sites) and MAF (11nt TGCTGAGTCAT; 2 sites). Moreover, all four TFs had predicted binding sites in the 2 kb sequence nearest the miR-302 cluster (Figure 6A). Maximum motif bit scores are given in Table 3, while the consensus binding sequence motifs are shown in Figure 6B. In addition, for GATA6 and MAF, we observed significant empirical *e*-values, calculated using the maximum motif bit score derived from predicted binding sites in the miR-302 promoter region (Table 3).

### Effect of relative miR-302 cluster over-expression in YSTs on global mRNA profiles

Messenger RNA gene regulation by microRNAs is principally determined by the microRNA 'seed' region, which binds to the seed complementary region (SCR) in the 3'-untranslated region (3'UTR) of mRNA targets [22]. The seed comprises nucleotides 2 to 8 of the microRNA, with nucleotides 2 to 7 (2-7nt) being most critical for mRNA binding specificity [8]. The four microRNAs miR-302a~302d share an identical 2-7nt seed region 'AAGUGC', corresponding to the SCR 'GCACTT'. We therefore tested for further enrichment of the common SCR GCACTT in the 3'UTRs of mRNAs differentially expressed in paediatric and adult YSTs versus germinomas. We analysed 16 paediatric samples (10 YST, six germinoma; Figure 1, Panel A) with matched microRNA and mRNA expression data, and the 20 suitable adult samples (eight YST, 12 germinoma) with published mRNA profiles [8]. For both paediatric and adult datasets there was enrichment of the common 2-7nt SCR GCACTT in genes significantly down-regulated in YSTs versus germinomas. Of down-regulated genes for which 3'UTR and transcript information was available, the SCR was present in 60/250 (24.0%) in the paediatric dataset and 58/243 (23.9%) in the adult dataset, significantly greater than the overall presence of the SCR in the genes on the array platform [2,125/13,042 (16.3%)] (*p *= 0.0012 paediatric, *p *= 0.0017 adult; two-tailed chi-squared test). Of the 60 SCR-containing mRNAs significantly down-regulated in paediatric YSTs versus germinomas, 34 were included in the equivalent adult list of 58 mRNAs, while 26 were seen for the paediatric dataset only. The 34 common mRNAs included apoptosis regulators (*CASP8, WDR33*), transcription factors (*PHTF2*) and integrin *ITGB2 *(Additional File 1, Table S2), while the 26 paediatric-only mRNAs included tumour suppressor genes (*RASSF2, BTG3*), additional apoptosis regulators (*RASSF2, PRKCB*), transcription factors (e.g. *TFEC*) and signal transducers (*RAB7L1, RAC2, SLC6A16*) (Additional File 1, Table S3).

## Discussion

Prognosis and clinical management vary considerably between the major histological subtypes of malignant GCT. We report, for the first time, the differences in global microRNA profiles identified by direct comparison of YSTs and germinomas arising across a broad range of patient ages and anatomical sites. Many of the top-ranking differentially expressed microRNAs are the same in the paediatric and adult datasets, consistent with the general observation that microRNA profiles reflect the developmental lineage of tumours [23]. We observed co-ordinate deregulation of members of particular microRNA clusters, namely miR-302 (including miR-367), miR-200a~200b and miR-200c/miR-141, in keeping with the finding that most microRNA clusters are regulated by a single promoter region.

Our data extend other reports. The miR-200 family and miR-205 are relatively under-expressed in germinomas, compared to YSTs and normal gonadal tissues, consistent with known roles for these microRNAs in preventing pluripotency [24] and with evidence that down-regulation (as seen for example in breast carcinomas [25] and mesothelioma [26]), is associated with epithelial to mesenchymal transition, tumour progression and metastasis [25,27-29]. Additionally, we show that miR-122, previously annotated as miR-122a, is over-expressed in all YSTs, not just those of the adult testis [30], and that miR-142-5p and miR-146a are over-expressed in all paediatric germinomas, not just intracranial tumours [31].

Of particular interest are members of the miR-302 cluster. We previously showed that microRNAs from the miR-302 and miR-371~373 clusters are co-ordinately over-expressed in all malignant GCTs [8]. In the present study we show further over-expression of the miR-302 cluster (but not miR-371~373) in YSTs versus germinomas, for both paediatric and adult tumours. In embryonic and germline stem cells, the miR-371~373 cluster is involved in maintaining the pluripotent state, whereas miR-302 members are induced during the first stages of *in vitro *differentiation [32]. As miR-302 is lost in cells and tissues showing somatic differentiation [33,34], it may be that levels peak during early extra-embryonic differentiation. If so, dynamic changes in miR-302 levels in normal embryonic development [35] would be mirrored in GCTs showing equivalent differentiation states, with high levels in tumours showing extra-embryonic differentiation [i.e. YSTs (yolk sac) and potentially choriocarcinoma (trophoblastic)] compared to undifferentiated tumours (germinomas); and a reduction to virtually undetectable levels in somatically differentiated tumours (teratomas), in which microRNA profiles are almost identical to normal gonadal tissues [8].

We addressed the cause of the increased miR-302 cluster expression in YSTs. We previously found no evidence of copy-number gain at the miR-302 genomic locus (4q25) in malignant GCTs of any type [8] and there are even reports of copy-number loss at this locus in paediatric intracranial YSTs [31]. These observations support other data showing that DNA copy number alterations account for only a minority of microRNA expression changes [36]. While miR-302 changes may be due to altered levels of TFs, pluripotency associated factors, such as *NANOG *and *POU5F1 (OCT3/4)*, which transcriptionally activate the miR-302 cluster promoter [21,37], are down-regulated in YSTs versus germinomas [7,38]. Accordingly, we identified 10 candidate TFs that are over-expressed in YSTs versus germinomas in both the paediatric and adult datasets and show positive correlations with miR-302 cluster expression levels. Of these, *GATA3 *and *GATA6*, markers of early endodermal differentiation, were previously identified as over-expressed in YSTs versus germinomas, using unsupervised analysis of global mRNA expression profiles [7], while others have identified YST over-expression of *GATA6 *and *PAX6 *(the latter a marker of neuro-ectodermal differentiation) [30].

Four of the 10 TFs have predicted binding sites in the miR-302 promoter region. Identification of TCF7L2 and MAF binding sites on the positive DNA strand, rather than the negative strand from which the miR-302 cluster is transcribed, is still compatible with an effect of these TFs on gene expression [39]. The six TFs without predicted binding sites may affect miR-302 cluster transcription through alternative mechanisms, such as long-range enhancer action (shown for *PAX6, PITX2, MAF *and members of the *SOX *gene family [40]) and/or association with other proteins that directly bind the miR-302 promoter. One further TF of potential relevance is *SALL4*, which has recently been shown to be a sensitive diagnostic marker of YSTs [41-43]. However, this gene was not represented on the microarrays used to generate the paediatric and adult mRNA expression datasets, nor was its corresponding binding motif available in the TRANSFAC database [20] used for iMotifs analysis.

We previously showed that the miR-302 cluster is over-expressed in all malignant GCTs (compared to normal gonad and benign GCTs), associated with co-ordinate down-regulation of a panel of mRNAs containing the 3'UTR SCR GCACTT, corresponding to the 2-7nt seed AAGUGC shared by miR-302a~302d [8]. Our present data indicate that the further miR-302 cluster over-expression seen in YSTs (regardless of patient age or anatomical site) causes down-regulation of other SCR-containing cancer-associated mRNAs, which may contribute substantially to the more aggressive clinical behaviour of YSTs compared to germinomas. Our observations also suggest that miR-302 family functions are concentration-dependent, with effects on some mRNA targets requiring the high expression levels seen in YSTs, and other effects occurring at the lower over-expression levels achieved in both germinomas and YSTs. Interestingly, similar concentration-dependent effects have recently been described for short interfering RNAs in mammalian cells [44].

## Conclusions

As well as providing insight into the biological differences between YSTs and germinomas, our data may contribute to further improvements in the clinical management of malignant GCTs. The robust discrimination between the two tumour types on global microRNA profiling was mirrored by our qRT-PCR findings, including in an independent test set that encompassed tumours from a broad range of anatomical sites. It will be interesting in future work to investigate the value of selected microRNAs as markers for improving malignant GCT diagnosis and as candidate targets for improving the treatment of tumours with adverse prognostic features.

## Competing interests

The authors declare that they have no competing interests.

## Authors' contributions

MJM participated in the study conception and design, performed experiments, analysed microarray and qRT-PCR data, interpreted all results and wrote the manuscript. HKS performed extensive data analysis, interpreted the results and wrote the manuscript. SvD performed data analysis and wrote the manuscript. RDP participated in the conception and design of the study, performed experiments and contributed to the manuscript. MP: undertook iMotifs analysis, interpreted the results and wrote the manuscript. BM was involved in the conception and design of the study and contributed to the manuscript. MRP was involved in the conception and design of the study and wrote the manuscript. CT provided pathological review and contributed to the manuscript. JCN participated in the conception and design of the study, provided clinical input and contributed to the manuscript. AJE provided bioinformatic advice, analysed data and contributed to the manuscript. NC: participated in the study conception and design, interpreted all results and wrote the manuscript. All authors read and approved the final manuscript.

## Supplementary Material

Additional file 1 **Additional Reference, Table S1, Table S2, Table S3**. Click here for file

Additional file 2 **Figure S1**. Click here for file
