# Supplementary material for: The two most common histological subtypes of malignant germ cell tumour are distinguished by global microRNA profiles, associated with differential transcription factor expression
Source: Mol Cancer. 2010 Nov 8;9:290. doi: 10.1186/1476-4598-9-290 (PMC2993676; doi:10.1186/1476-4598-9-290)
Supplement: Additional file 1 — Additional Reference, Table S1, Table S2, Table S3. [file 1476-4598-9-290-S1.DOCX]

**Additional Data**

**Additional Reference**

1. Steinbiss S, Gremme G, Scharfer C, Mader M, Kurtz S: **AnnotationSketch: a genome annotation drawing library.** *Bioinformatics* 2009, **25:**533-534.

**Table S1.** **Predicted transcription factor binding sites in the miR-302 cluster promoter region, obtained using the sequence analysis environment iMotifs.** A total of 41 binding sites for GATA6, GATA3, TCF7L2 and MAF were identified in the 10kb upstream sequence from the last member of the miR-302 cluster, miR-367. For each site, the Table gives the DNA strand binding, location within the 10kb upstream sequence, exact genomic location (<http://www.ensembl.org/>) and associated motif bit scores. In iMotifs the optimal bit score is normalised to zero, with all other bit scores having negative values. Accordingly, the closer a score to zero, the greater is the significance of the predicted transcription factor binding site.

| **Transcription**  **Factor** | **Length (bp)** | **DNA**  **Strand** | **Start in Upstream 10kb Sequence** | **Stop in Upstream 10kb**  **Sequence** | **Ensembl**  **Start** | **Ensembl**  **Stop** | **Motif Bit Score** |
| --- | --- | --- | --- | --- | --- | --- | --- |
| GATA6 | 10 | Negative | 111 | 120 | 113569141 | 113569150 | -1.84809428 |
| GATA6 | 10 | Negative | 1104 | 1113 | 113570134 | 113570143 | -1.077995672 |
| GATA6 | 10 | Positive | 1283 | 1292 | 113570313 | 113570322 | -1.722091612 |
| GATA6 | 10 | Positive | 2578 | 2587 | 113571608 | 113571617 | -1.930557741 |
| GATA6 | 10 | Negative | 2786 | 2795 | 113571816 | 113571825 | -1.494458802 |
| GATA6 | 10 | Positive | 3444 | 3453 | 113572474 | 113572483 | -1.213700592 |
| GATA6 | 10 | Positive | 3461 | 3470 | 113572491 | 113572500 | -1.57298969 |
| GATA6 | 10 | Positive | 3670 | 3679 | 113572700 | 113572709 | -1.84809428 |
| GATA6 | 10 | Positive | 3712 | 3721 | 113572742 | 113572751 | -1.340477834 |
| GATA6 | 10 | Negative | 3910 | 3919 | 113572940 | 113572949 | -1.710495645 |
| GATA6 | 10 | Negative | 4403 | 4412 | 113573433 | 113573442 | -1.855609539 |
| GATA6 | 10 | Negative | 6584 | 6593 | 113575614 | 113575623 | -1.715672316 |
| GATA6 | 10 | Negative | 6595 | 6604 | 113575625 | 113575634 | -1.423968151 |
| GATA6 | 10 | Positive | 6598 | 6607 | 113575628 | 113575637 | -1.333996904 |
| GATA6 | 10 | Negative | 6644 | 6653 | 113575674 | 113575683 | -1.382855268 |
| GATA6 | 10 | Negative | 6660 | 6669 | 113575690 | 113575699 | -0.514095006 |
| GATA6 | 10 | Negative | 6703 | 6712 | 113575733 | 113575742 | -0.27563467 |
| GATA6 | 10 | Negative | 8693 | 8702 | 113577723 | 113577732 | -1.011593201 |
| GATA6 | 10 | Negative | 9390 | 9399 | 113578420 | 113578429 | -0.806826026 |
|  | | | | | | | |
| TCF7L2 | 8 | Positive | 345 | 352 | 113569375 | 113569382 | -0.584963943 |
| TCF7L2 | 8 | Positive | 7316 | 7323 | 113576346 | 113576353 | -0.584963943 |
|  | | | | | | | |
| MAF | 11 | Positive | 522 | 532 | 113569552 | 113569562 | -5.45121388 |
| MAF | 11 | Positive | 2492 | 2502 | 113571522 | 113571532 | -3.925995248 |
|  | | | | | | | |
| GATA3 | 9 | Negative | 112 | 120 | 113569142 | 113569150 | -3.657376347 |
| GATA3 | 9 | Positive | 889 | 897 | 113569919 | 113569927 | -3.516020574 |
| GATA3 | 9 | Positive | 1082 | 1090 | 113570112 | 113570120 | -1.958182498 |
| GATA3 | 9 | Negative | 1105 | 1113 | 113570135 | 113570143 | -3.501257145 |
| GATA3 | 9 | Positive | 1156 | 1164 | 113570186 | 113570194 | -3.336694121 |
| GATA3 | 9 | Positive | 1283 | 1291 | 113570313 | 113570321 | -3.378517051 |
| GATA3 | 9 | Positive | 1865 | 1873 | 113570895 | 113570903 | -2.421583018 |
| GATA3 | 9 | Negative | 2100 | 2108 | 113571130 | 113571138 | -3.595262752 |
| GATA3 | 9 | Negative | 2655 | 2663 | 113571685 | 113571693 | -2.623274454 |
| GATA3 | 9 | Positive | 3240 | 3248 | 113572270 | 113572278 | -2.629359374 |
| GATA3 | 9 | Positive | 3461 | 3469 | 113572491 | 113572499 | -1.734541611 |
| GATA3 | 9 | Negative | 6645 | 6653 | 113575675 | 113575683 | -1.76553742 |
| GATA3 | 9 | Negative | 6661 | 6669 | 113575691 | 113575699 | -3.55254645 |
| GATA3 | 9 | Negative | 6666 | 6674 | 113575696 | 113575704 | -3.770831643 |
| GATA3 | 9 | Negative | 6730 | 6738 | 113575760 | 113575768 | -2.465977138 |
| GATA3 | 9 | Negative | 8831 | 8839 | 113577861 | 113577869 | -2.757023247 |
| GATA3 | 9 | Positive | 9212 | 9220 | 113578242 | 113578250 | -3.937484343 |
| GATA3 | 9 | Negative | 9391 | 9399 | 113578421 | 113578429 | -3.851757066 |

**Table S2.** **Down-regulated gene targets of miR-302a~302d in paediatric YSTs versus germinomas.** Together, Tables S2 and S3 list the 60 genes significantly down-regulated in paediatric YSTs versus paediatric germinomas for which the 3’UTR contains the SCR corresponding to the common 2-7nt seed of miR-302a~302d. Table S2 gives the 34 genes that were also present in the list of 58 genes significantly down-regulated in adult YSTs (versus adult germinomas), in which the common SCR GCACTT was present in the 3’UTR. Table S3 lists the remaining 26 genes that were significantly down-regulated in the paediatric YSTs only.

| **Gene Name** | **Paediatric**  **Rank** | **Paediatric**  **Log_2_ Fold Change** | **Adult**  **Rank** | **Adult**  **Log_2_ Fold**  **Change** | **Function**  **ENTREZ GENE:** [**http://www.ncbi.nlm.nih.gov/gene/**](http://www.ncbi.nlm.nih.gov/gene/) **and**  **GENATLAS** [**http://genatlas.medecine.univ-paris5.fr/**](http://genatlas.medecine.univ-paris5.fr/) **)** |
| --- | --- | --- | --- | --- | --- |
| ***TFAP2C*** | **1** | -4.06 | **1** | -4.12 | Transcription factor; involved in the activation of several developmental genes |
| ***WDR33*** | **2** | -4.04 | **2** | -3.70 | Involved in cell cycle progression, signal transduction, apoptosis, and gene regulation. Highly expressed in normal testis |
| ***GABBR1*** | **3** | -3.45 | **10** | -2.72 | GABA B receptor 1 |
| ***CPEB1*** | **4** | -3.27 | **4** | -3.27 | Cytoplasmic polyadenylation element (CPE) binding protein 1; binds CPE in 3' UTR of some mRNAs; regulates cyclin B1 translation in embryonic cell divisions |
| ***SLAMF8*** | **5** | -3.18 | **32** | -1.86 | CD2 family of cell surface proteins; lymphocyte activation |
| ***ETV1*** | **6** | -3.10 | **3** | -3.6 | Transcription factor; member of ETS family: ETS variant 1 |
| ***UCHL1*** | **7** | -2.87 | **39** | -1.78 | Ubiquitin carboxyl-terminal esterase L1; peptidase C12 family |
| ***ITGB2*** | **8** | -2.82 | **27** | -1.95 | Integrin cell-surface protein; cell adhesion and cell-surface mediated signaling. Also termed CD18 |
| ***NUDT11*** | **9** | -2.65 | **7** | -3.06 | Phospho-hydrolase |
| ***ELOVL6*** | **10** | -2.55 | **55** | -1.55 | Long-chain fatty-acyl elongase; hepatic lipogenesis |
| ***PHTF2*** | **12** | -2.50 | **8** | -2.96 | Transcription factor |
| ***OSBPL3*** | **13** | -2.49 | **35** | -1.81 | Oxysterol-binding protein (OSBP) family, a group of intracellular lipid receptors |
| ***NEFH*** | **14** | -2.41 | **5** | -3.24 | Neurofilament heavy polypeptide; component of axoskeleton |
| ***TBL1X*** | **15** | -2.27 | **23** | -2.05 | Transducin, beta-like, 1X; involved in protein-protein interactions |
| ***SACS*** | **16** | -2.26 | **42** | -1.72 | Sacsin protein; highly expressed in the central nervous system |
| ***MKRN1*** | **18** | -2.21 | **20** | -2.14 | Makorin ring finger protein-1 gene |
| ***PRNP*** | **19** | -2.21 | **14** | -2.36 | Glycosyl-phosphatidyl-inositol-anchored glycoprotein |
| ***RPS6KA5*** | **20** | -2.12 | **22** | -2.06 | Ribosomal protein S6 kinase polypeptide 5 |
| ***IL6R*** | **21** | -2.10 | **12** | -2.59 | IL6 receptor; dysregulated in prostate cancer |
| ***KIAA0226*** | **23** | -1.97 | **47** | -1.65 | Unknown |
| ***STOM*** | **24** | -1.96 | **41** | -1.73 | Stomatin; erythrocyte membrane protein band 7.2 |
| ***CD47*** | **27** | -1.92 | **6** | -3.06 | Membrane protein; increases intracellular Ca++ upon cell adhesion to extracellular matrix |
| ***CASP8*** | **29** | -1.92 | **34** | -1.81 | Caspase 8; apoptosis; mutations in bladder cancer |
| ***LMO2*** | **31** | -1.88 | **9** | -2.74 | LIM-domain protein; yolk sac erythropoiesis |
| ***IL10RA*** | **32** | -1.85 | **58** | -1.50 | IL10 receptor A, inhibits production of pro-inflammatory cytokines |
| ***PRKAR2B*** | **34** | -1.77 | **11** | -2.71 | cAMP dependent protein kinase |
| ***LDLRAP1*** | **39** | -1.72 | **45** | -1.69 | Low density lipoprotein receptor adaptor protein 1 |
| ***MMP2*** | **42** | -1.70 | **33** | -1.84 | Matrix metalloproteinase protein 2 (MMP) family |
| ***FGL2*** | **43** | -1.69 | **48** | -1.63 | Fibrinogen-like 2; mucosal physiological functions |
| ***MICB*** | **44** | -1.69 | **18** | -2.19 | NKG2D II receptor ligand; activates NK & CD8+ T cells |
| ***INPP5F*** | **48** | -1.60 | **19** | -2.17 | Inositol polyphosphate-5-phosphatase F |
| ***TRIM2*** | **50** | -1.58 | **29** | -1.87 | Tripartite motif family; localizes to cytoplasmic filaments |
| ***ATG5*** | **51** | -1.58 | **44** | -1.69 | ATG5 autophagy related 5 homolog; involved in apoptosis |
| ***KIAA0922*** | **53** | -1.56 | **52** | -1.60 | Unknown |

**Table S3.** **Down-regulated gene targets of miR-302a~302d in paediatric YSTs versus germinomas.** Together, Tables S2 and S3 list the 60 genes significantly down-regulated in paediatric YSTs versus paediatric germinomas for which the 3’UTR contains the SCR corresponding to the common 2-7nt seed of miR-302a~302d. Table S2 gives the 34 genes that were also present in the list of 58 genes significantly down-regulated in adult YSTs (versus adult germinomas), in which the common SCR GCACTT was present in the 3’UTR. Table S3 lists the remaining 26 genes that were significantly down-regulated in the paediatric YSTs only.

| **Gene Name** | **Paediatric**  **Rank** | **Paediatric**  **Log_2_ Fold**  **Change** | **Function**  **ENTREZ GENE:** [**http://www.ncbi.nlm.nih.gov/gene/**](http://www.ncbi.nlm.nih.gov/gene/) **and**  **GENATLAS** [**http://genatlas.medecine.univ-paris5.fr/**](http://genatlas.medecine.univ-paris5.fr/) **)** |
| --- | --- | --- | --- |
| ***CTSS*** | **11** | -2.53 | Cathepsin S; lysosomal cysteine protease; degrades proteins to peptides for presentation to MHC Class II molecules |
| ***ETV5*** | **17** | -2.26 | ETS variant gene 5; transcription factor; involved in spermatogonial stem cell self-renewal |
| ***SOD2*** | **22** | -2.06 | Mitochondrial manganese superoxide dismutase 2; antioxidant/free radical defence. Polymorphisms of this gene associated with increased cancer risk |
| ***NRCAM*** | **25** | -1.94 | Neuronal cell adhesion molecule |
| ***TFEC*** | **26** | -1.93 | Transcription factor EC |
| ***EEF1B1*** | **28** | -1.92 | Eukaryotic translation elongation factor 1 beta 1 |
| ***NCF2*** | **30** | -1.88 | Neutrophil cytosolic factor 2; involved in immunity/defence |
| ***IL22RA1*** | **33** | -1.79 | IL22 receptor |
| ***GZMK*** | **35** | -1.76 | Granzyme K; a lymphocyte tryptase member of related serine proteases |
| ***TBC1D8*** | **36** | -1.76 | TBC1 domain family 8; GTPase activator |
| ***SHROOM2*** | **37** | -1.74 | Shroom family member 2; believed to be involved in control of actin cytoskeleton |
| ***KMO*** | **38** | -1.74 | Kynurenine 3-mono-oxygenase |
| ***RASSF2*** | **40** | -1.72 | Tumour suppressor gene; Ras association domain family 2; promotes apoptosis and cell cycle arrest |
| ***KIT*** | **41** | -1.71 | Tyrosine protein kinase (CD117) |
| ***RAB7L1*** | **45** | -1.68 | RAB7, member Ras-oncogene family-like 1; signal transduction |
| ***BTG3*** | **46** | -1.68 | Tumour suppressor gene; transcript regulation, G2/M checkpoint modulator |
| ***GOLGA9p*** | **47** | -1.63 | Golgi autoantigen, pseudogene 9 |
| ***MBP*** | **49** | -1.59 | Myelin basic protein; a structural protein |
| ***PIK3R3*** | **52** | -1.57 | Phospho-inositide-3-kinase regulatory subunit 3; involved in signalling |
| ***PRKCB*** | **54** | -1.56 | Protein kinase C, beta-1; involved in apoptosis induction |
| ***ALOX15B*** | **55** | -1.55 | Arachidonate 15-lipoxygenase type 2; leukotriene production |
| ***KIF14*** | **56** | -1.53 | Kinesin family member 14; involved in cell division |
| ***MYB*** | **57** | -1.53 | Myeloblastosis viral oncogene homolog; a transcription factor involved in cell proliferation and implicated in tumorigenesis |
| ***AHI-1*** | **58** | -1.52 | Abelson helper integration site; mediates protein:protein interactions |
| ***RAC2*** | **59** | -1.52 | GTPase; involved in signal transduction |
| ***SLC6A16*** | **60** | -1.52 | Solute carrier family 6, member 16; involved in signal transduction |
