# Supplementary material for: The two most common histological subtypes of malignant germ cell tumour are distinguished by global microRNA profiles, associated with differential transcription factor expression
Source: Mol Cancer. 2010 Nov 8;9:290. doi: 10.1186/1476-4598-9-290 (PMC2993676; doi:10.1186/1476-4598-9-290)
Supplement: Additional file 2 — Figure S1. [file 1476-4598-9-290-S2.PPTX]

## Slide 1
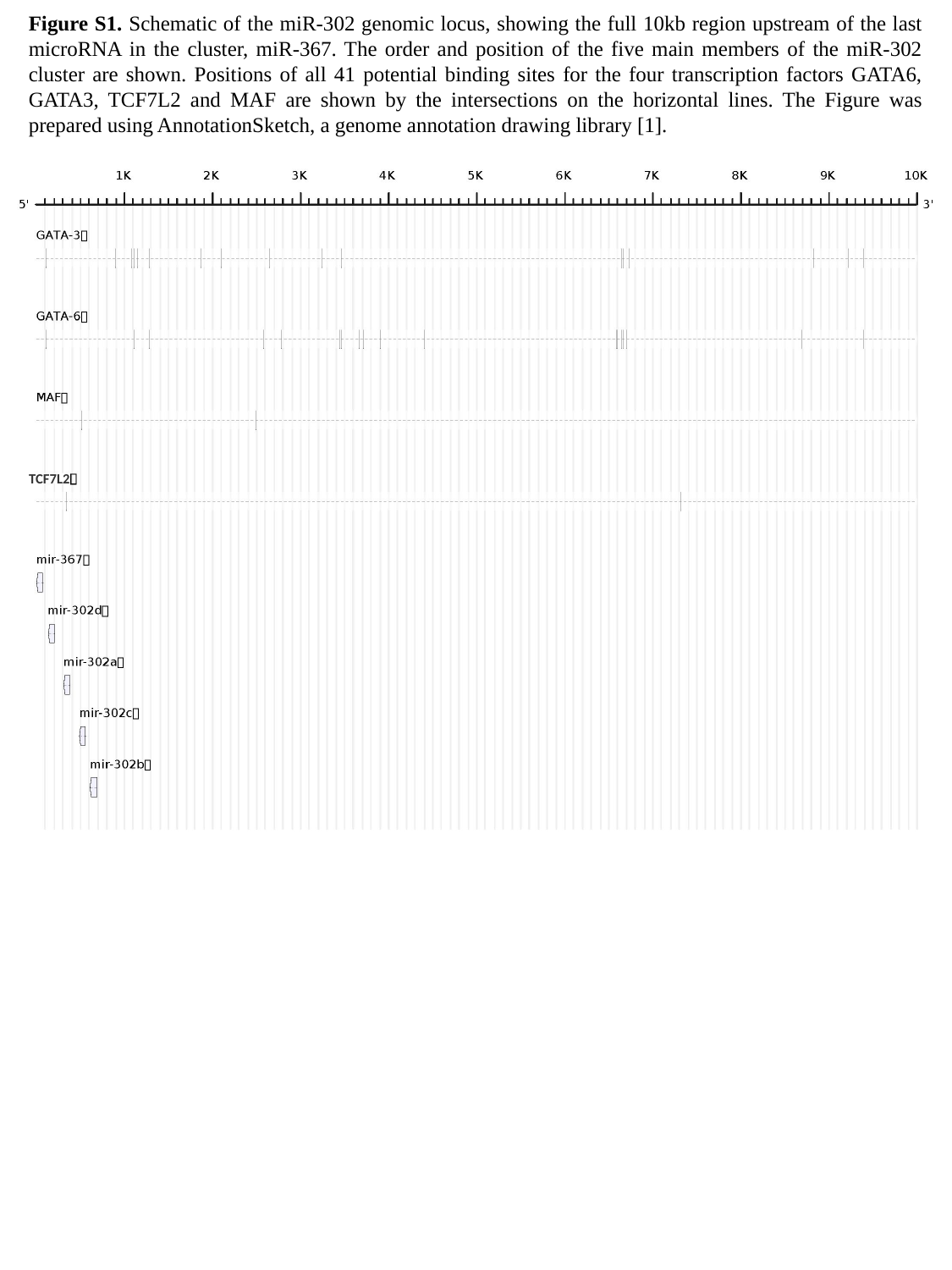

Figure S1. Schematic of the miR-302 genomic locus, showing the full 10kb region upstream of the last microRNA in the cluster, miR-367. The order and position of the five main members of the miR-302 cluster are shown. Positions of all 41 potential binding sites for the four transcription factors GATA6, GATA3, TCF7L2 and MAF are shown by the intersections on the horizontal lines. The Figure was prepared using AnnotationSketch, a genome annotation drawing library [1].
TCF7L2
